# Effect of shaking and vibration stimulation on lumbar vertebrae in ovariectomized mice

**DOI:** 10.20407/fmj.2018-006

**Published:** 2019-04-17

**Authors:** Takumi Kito, Kazuhiro Nishii, Runhong Yao, Toshio Teranishi, Tomohisa Sugiyama, Kazuyoshi Sakai, Mamoru Matsubara, Kouji Yamada

**Affiliations:** 1 Graduate School of Health Sciences, Fujita Health University, Toyoake, Aichi, Japan; 2 Department of Bioscience and Biotechnology, Kyoto Gakuen University, Kyoto, Japan

**Keywords:** Ovariectomized (OVX) mice, Bone density, Vibration stimulation, Shaking stimulation

## Abstract

**Objectives:**

Bone fractures affect the activities of daily living and lower quality of life, so investigating preventative measures is important. We developed novel stimulation equipment that combined a vibration stimulus with a shaking stimulus for preventing osteoporosis (one of the causes of bone fractures). We aimed to investigate the effect of this equipment on ovariectomized mice.

**Methods:**

Oophorectomy of 8-week-old female mice was done. The stimulation group was stimulated for 10 consecutive weeks.

**Results:**

The stimulation group showed significantly higher values (p<0.05) for osteoid thickness, osteoid volume-to-bone volume ratio and mineral apposition rate than those in the non-stimulation control group. The stimulation group showed significantly higher values (p<0.05) compared with the non-stimulation for expression of bone morphogenetic protein-2, interleukin-1β, interleukin-6 and myogenic determination gene in quadriceps femoris muscles (QFMs).

**Conclusions:**

These data suggest that cytokine secretion by QFMs carried a humoral factor throughout the body *via* the blood and blood vessels and acted on bone and various organs. Development of this stimulation method and its clinical application, new methods for preventing and treating osteoporosis could ensue.

## Introduction

Major reasons for caregiving of older people in Japan include avoidance of cerebrovascular disease, dementia and bone fractures.^1^ At present, appropriate habitual exercises, such as walking, are strongly recommended to prevent these diseases. Exercise is believed to prevent reductions in (i) the muscle strength required for daily life and (ii) bodily functions (e.g., circulation and respiration). In addition, spending time outside is related to mental health. However, detailed analyses of the effect on bone quality due to activities of daily life (ADL) are scarce. Therefore, we focused on methods of exercise stimulation and their effects on bone.

Osteoporosis is a disease that typically affects older women. It is said to be caused by a reduction in estrogen levels after the menopause. Osteoporosis confers a high risk of bone fracture, which is difficult to treat in older people, affecting ADL and reducing quality of life. To avoid osteoporosis, preventative measures should be considered.

The most likely sites of bone fractures accompanying osteoporosis include compression fractures of the femoral neck and lumbar vertebrae. Fractures of lumbar vertebrae can be due to external unforeseen events (e.g., falls) but are possible even during ADL (e.g., sitting down). There has been much research into femoral-head fractures and exercise stimulation to increase bone density in older people. Bassey and Ramsdale reported that femur density increased more in one group of female patients that undertook high-strength exercise (jumping and skipping) habitually for 1 year than in another group of female patients that carried out low-strength exercise (bending and stretching of legs) habitually.^2^

Compared with research on the femur, studies on lumbar vertebrae have been scarce. Exercises such as jumping and running are considered to be difficult for an older person whose body condition has deteriorated. Therefore, a recommended exercise method to increase bone density to prevent compression fractures of lumbar vertebrae in older people that is safe and considers the body’s capabilities is lacking.

In exercise-related research using experimental animals, methods that have been selected are treadmills, forced squatting through electrical stimulation, and exercise stimulation through swimming equipment. However, these exercises have been used as methods for making animals exercise without investigating the effect on muscular hypertrophy or bone. In addition, these studies have been limited because: the equipment requires extensive setup time and effort before, during, and after the experiment; the equipment is expensive; and it is difficult to exercise several animals simultaneously. In the present study, we used experimental animals for which an effective exercise-stimulation method for muscles and bones had not been established, and novel stimulation equipment was employed to analyze the effect on bone and muscle.

The novel equipment used in our research combined vibration and shaking for stimulation. Roelants and colleagues stated that with vibration stimulation, a change is seen that is similar to the change observed after resistance training (which is a standard method for providing an increase in muscle strength in older women).^3^ Blottner and co-workers stated that with vibration stimulation, during treatment in bed for 8 weeks, the structure and strength generation in the gastrocnemius muscle were maintained.^4^ Therefore, vibration stimulation is believed to maintain muscle strength. Manske and colleagues postulated that muscle contraction, as an element of direct physical stimulation, is necessary to increase bone density.^5^

Consideration has been given to forced muscle contraction by a vibration stimulus applied as a direct physical stimulus to the femur or lumbar vertebrae. The shaking stimulus is circular motion of a flat plate whose axis of rotation is displaced. Scholars have reported^6–9^ that administration of a shaking stimulus on mouse models of osteoporosis mitigates rapid reduction in bone density in the femur and lumbar vertebrae. In a shaking stimulus, it is thought that displacement of the flat plate promotes the “balancing operation” that activates muscles throughout the body to maintain posture and avoid falling. It is thought that this exercise induces a knee reflex and creates “isometric contraction”, which encourages continuous muscle contraction and helps prevent falling. It has also been reported^10^ reported that combination of a vibration stimulus and shaking stimulus mitigates inflammation and slows arthritis induction in mice. Based on the results mentioned above we conjectured that, through combination of a vibration stimulus and shaking stimulus, forced muscle contraction and the accompanying direct stimulus to the bone would have a synergistic effect on mitigating a reduction in bone density.

## Materials and Methods

### Ethical approval of the study

This research was undertaken based on the Guidelines for Animal Experimentation set by Fujita Health University (H0702; Aichi, Japan).

### Experimental animals

We used 12 female mice (8 weeks; CLEA Japan, Tokyo, Japan) and raised them in groups in cages with floor mats (3 animals per cage). Oophorectomy was undertaken after 1 week of acclimatization to create ovariectomized (OVX) mice, which have reduced bone density. Stimulation was carried out after 1 week.

### Creation of an osteoporosis model in mice

After the induction of general anesthesia (isoflurane), we undertook incision of the skin and muscle of the lower back in mice. The ovary for each fallopian tube was exposed. We removed each ovary attached to a fallopian tube, and then hemostasis was undertaken. After removing both ovaries, the wound was sutured.

### Stimulation method

**Figure 1:**
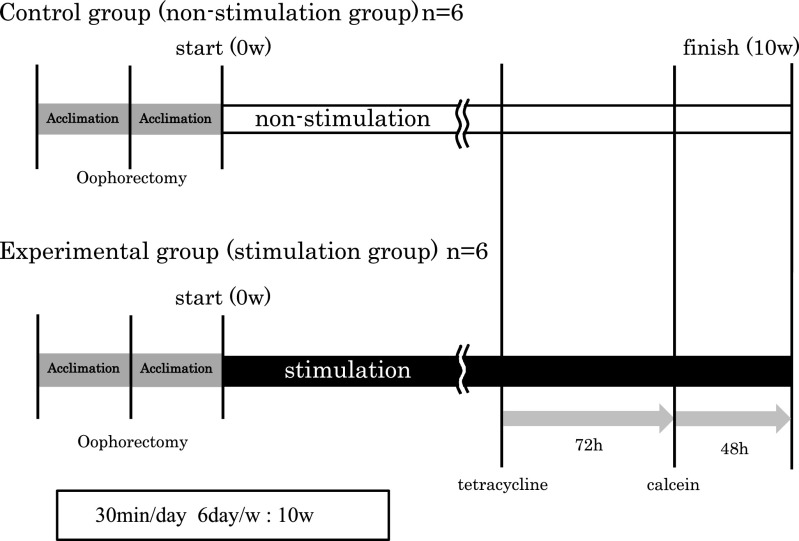
Experiment protocol Mice were divided into two groups of six: stimulation and non-stimulation (control). For the stimulation group, after 1 week of acclimatization from oophorectomy, we undertook stimulation once each day for 30 min, 6 days in a row, followed by 1 rest day for 10 consecutive weeks.

Mice were divided into two groups of six: stimulation and non-stimulation (control). For the stimulation group, after 1 week of acclimatization from oophorectomy, we undertook stimulation once each day, for 30 min, over 6 days in a row followed by 1 day of rest, for 10 consecutive weeks. For the non-stimulation (control) group, after 1 week of acclimatization from oophorectomy, we raised them normally without stimulation (Figure 1). The environmental conditions for raising mice were identical for both groups.

### Stimulation equipment

**Figure 2:**
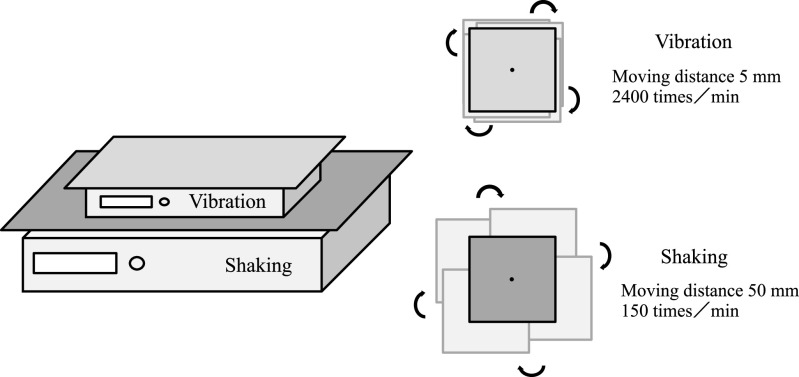
Stimulation equipment A horizontal rotation device that can administer uniform 360° horizontal shaking stimulations at 150 times/min (NX-25D; Nissin Scientific Corporation, Tokyo, Japan) is shown. The other device is a vibrator that can administer 5-mm tornado-type vibrations at 2400 times/min (SK-40-D1; Nissin Scientific Corporation). These stimulation devices were combined into a “vibration and shaking” device.

We commissioned stimulation equipment from Nissin Scientific Corporation (Tokyo, Japan). This tailor-made equipment combined moving-axis, flat-rotation shaking equipment (NX-25D; Nissin Scientific Corporation) that gives a homogeneous stimulus from any horizontal direction up to 360°, and vibration equipment (SK-40-D1; Nissin Scientific Corporation) with a movement distance of 5 mm. We set the shaking stimulation to 150 times/min and vibration stimulation to 2400 times/min (Figure 2).

### Double-labeling of bone

To carry out osteomorphometry during tissue analyses, we labeled bone with tetracycline (Merck, Darmstadt, Germany) and calcein (Dojindo Molecular Technologies, Tokyo, Japan). We adjusted the amount of tetracycline with phosphate-buffered saline (PBS) to a solution of 2 mg/mL. We dissolved 0.5 g of calcein in 2 mL of KOH solution (1 N), adjusted the pH to 7.0 with NaOH, added 25 mL of PBS, and placed it into a light-shielding bottle for temporary storage. Just before use, we added 8 mL of PBS to 2 mL of this solution to adjust it to 2 mg/mL. Furthermore, 96 h before collecting a sample, we injected tetracycline (0.4 mL/40 g body weight, s.c.) into the back of the mouse. Then, 48-h later, we injected calcein (0.4 mL/40 g body weight, s.c.) into the same site.

### Creation of bone-tissue samples

**Figure 3:**
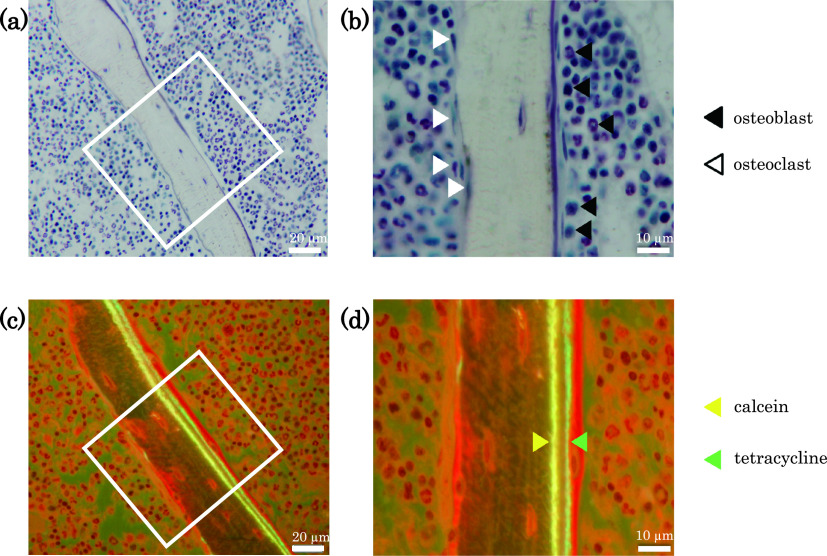
Villanueva Osteochrome Bone Stain of lumbar vertebrae Lumbar vertebrae stained with Villanueva Osteochrome Bone Stain. Images show sagittal cross-sections of lumbar vertebrae of osteoporotic model mice stained with Villanueva Osteochrome Bone Stain. (a) and (c) show low-power fields (scale bar=20 μm). (b) and (d) show high-power fields (scale bar=10 μm). (a) and (b) were observed under natural light. (c) and (d) were observed under fluorescent light. Black arrowhead=osteoblasts; white arrowhead=osteoclasts; green arrowhead=calcein; yellow arrowhead=tetracycline.

After the final stimulation, and after the induction of under general anesthesia (isoflurane), we opened the chests of mice. We irrigated the entire body with PBS, extracted lumbar vertebrae, and fixed them in 70% ethanol. Once a day, we replaced 70% ethanol with fresh 70% ethanol thrice. After fixing, we removed the muscle and soft tissue and immersed them in Villanueva Osteochrome Bone Stain solution for ~4 days. Then, we immersed them in 70% ethanol for 30 min, 95% ethanol for 30 min, 100% ethanol for 30 min, and acetone for 30 min to remove water. After removing water, we immersed the sample for 24 h in a mixed solution of 3 parts of methylmethacrylate (MMA) monomer to 1 part of acetone. Then, we prepared MMA resin by mixing 42 g of polymethylmethacrylate beads and 1.65 g of benzoyl peroxide into 125 mL of MMA monomer, and embedded the sample. We polymerized the embedded sample in an incubator for 10 days while increasing the temperature gradually from 30°C to 40°C. We sliced the tissue sample with a microtome to a thickness of 5 μm, and encapsulated it using a photocuring encapsulating agent to create a tissue sample. We calculated the primary parameters such as osteoid volume (OV), osteoid surface (OS), osteoid thickness (O.Th), osteoblast number (N.Ob) and multicellular osteoclast number (N.Mu.Oc). Furthermore, the secondary parameters were calculated from the primary parameters. We calculated the bone volume-to-tissue volume ratio (BV/TV), trabecular thickness (Tb.Th), trabecular number (Tb.N) and trabecular separation (Tb.Sp) as parameters that express bone volume; osteoid volume-to-bone volume ratio (OV/BV) as a parameter that expresses bone formation; and mineral apposition rate (MAR) and bone formation rate-to-tissue volume ratio (BFR/TV) as parameters that express calcification (Figure 3).

### Creation of a solution of protein from quadriceps femoris muscles (QFMs)

After the final stimulation, after the induction of general anesthesia (isoflurane), we opened the chests of mice. We irrigated the entire body with PBS, collected QFMs, froze them rapidly with dry ice, and stored them temporarily at –80°C. Then, we pulverized the frozen QFMs using a mortar under liquid nitrogen, and dissolved them in T-PER (PB196592; Thermo Scientific, Waltham, MA, USA), which contains a protease inhibitor (Complete Protease Inhibitor Cocktail; 10190300; Roche, Basel, Switzerland). After centrifugation (5000 rpm, 5 min), we collected the supernatant, adjusted it to a final concentration of 1 μg/μL with T-PER, and stored it at –80°C.

### Protein expression

We measured levels of interleukin (IL)-6, IL-15, IL-8 and IL-1β using a Mouse IL-6 Quantikine ELISA Kit (R&D Systems, Inc. USA), Mouse IL-15 ELISA Kit (Abcam plc, Cambridge, UK), Mouse CXCL2/MIP-2 Quantikine ELISA Kit (R&D Systems, Inc. USA) and Mouse IL-1 beta ELISA Kit (Abcam plc, Cambridge, UK). For analyses of muscular hypertrophy, we undertook western blotting using antibody against the myogenic determination (MyoD) gene (H2107; Santa Cruz Biotechnology, Santa Cruz, CA, USA) and measured the detected bands with ImageJ (National Institutes of Health, Bethesda, MD, USA).

### Statistical analyses

We carried out statistical analyses to compare bone histomorphometry and protein expression from QFMs between the non-stimulation group and stimulation group using the Student’s *t*-test. SPSS v22.0 (IBM, Armonk, NY, USA) was used for analyses and p<0.05 (two-tailed) considered significant.

## Results

**Figure 4:**
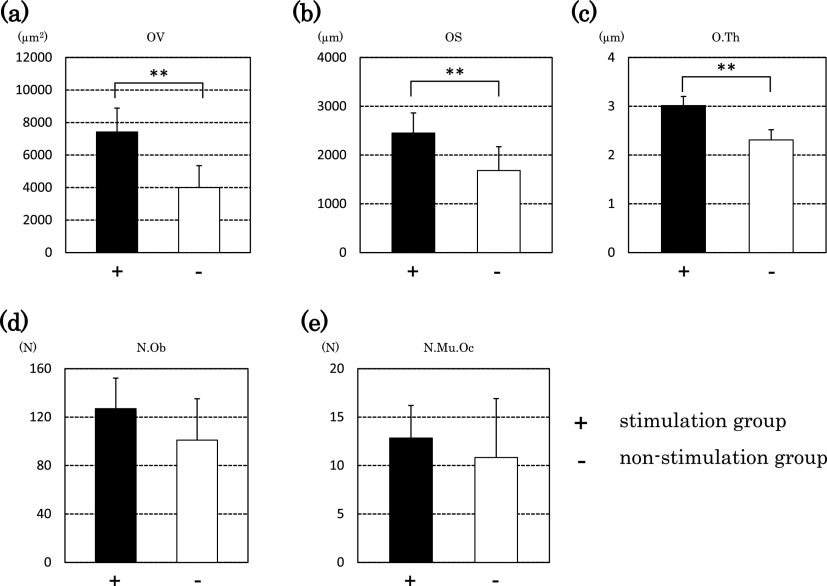
Bone histomorphometry (primary parameters) Parameters of bone structure are shown. The graphs show results for osteoid volume (OV), osteoid surface (OS), osteoid thickness (O.Th), osteoblast number (N.Ob) and multinuclear osteoclast number (N.Mu.Oc). Black bars represent the stimulation group (+) and white bars represent the non-stimulation group (–). Significant differences were observed (**p<0.01). The number of animals in each group was six.

**Figure 5:**
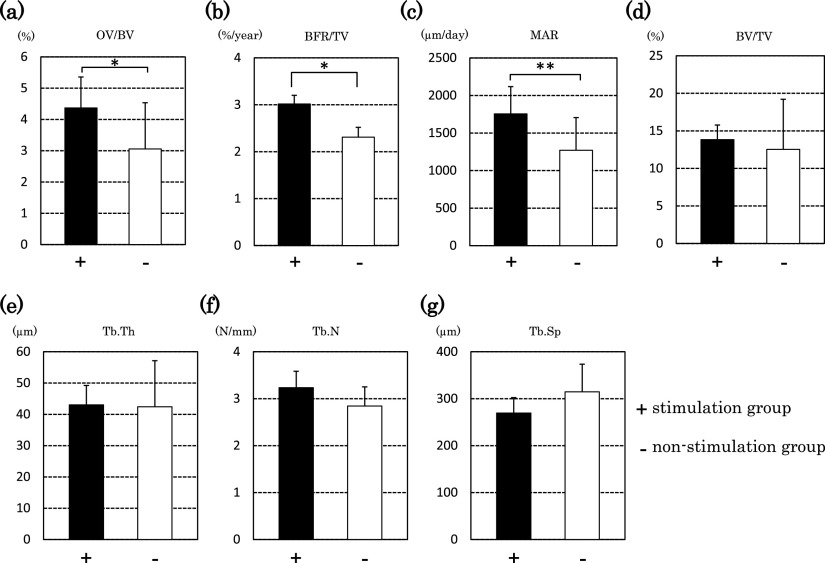
Bone histomorphometry (secondary parameters) Parameters of bone structure are shown. The graphs show results for bone volume-to-tissue volume (BV/TV) ratio, trabecular thickness (Tb.Th), trabecular number (Tb.N), trabecular separation (Tb.Sp), osteoid volume-to-bone volume (OV/BV) ratio, mineral apposition rate (MAR) and bone formation rate-to-tissue volume (BFR/TV) ratio. Black bars represent the stimulation group (+) and white bars represent the non-stimulation group (–). Significant differences were observed (**p<0.01, *p<0.05). The number of animals in each group was six.

The stimulation group showed significantly higher values for OV, OS, O.Th, MAR, OV/BV and BFR/TV than those in the non-stimulation group (p<0.01 and p<0.05) (Figures 4 and 5). We did not find a significant difference for N.Ob or Tb.N, but the stimulation group tended to have higher values than those in the non-stimulation group (p=0.082 and 0.053, respectively) (Figures 4 and 5). We did not find a significant difference for Tb.Sp, but the stimulation group tended to show a lower value than that in the non-stimulation group (p=0.067) (Figure 5).

**Figure 6:**
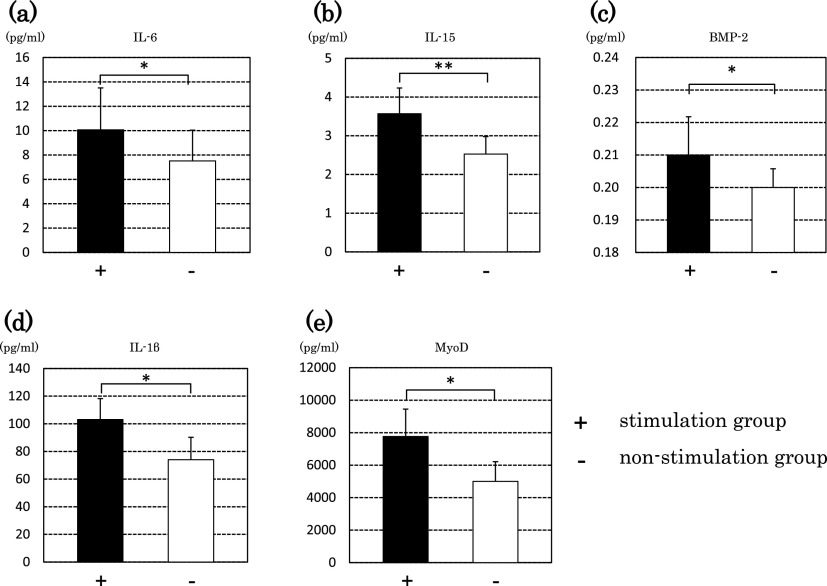
Protein expression in QFMs Graphs show ELISA results for (a) IL-6, (b) IL-15, (c) BMP-2, (d) IL-1β and (e) MyoD. Black bars represent the stimulation group (+) and white bars represent the non-stimulation group (–). Significant differences were observed (**p<0.01, *p<0.05).

We created a solution of QFMs and measured expression of bone morphogenetic protein (BMP)-2, IL-1β, IL-6 and IL-15 using ELISAs. The stimulation group showed significantly higher values than those in the non-stimulation group for BMP-2, IL-1β, IL-6 and IL-15 (p<0.01 and p<0.05) (Figure 6). Measurement of MyoD expression by western blotting demonstrated that the stimulation group showed a significantly higher value than that in the non-stimulation group (p<0.05).

## Discussion

We created equipment that carried out two types of stimulation simultaneously: shaking and vibration. This equipment was used on OVX mice, and we undertook a comparative study on bone and muscle.

For OS, O.Th and OV, which are primary parameters for osteomorphometry, the stimulation group showed higher values than those in the non-stimulation group. For one secondary parameter, the OV/BV, the stimulation group showed a significantly higher value than that in the non-stimulation group. For MAR and the BFR/TV, the stimulation group showed significantly higher values than those in the non-stimulation group. These results suggest that the stimulation equipment promoted an increase in the osteoid cells needed for bone formation, as well as promoting mineral apposition simultaneously; thus, bone formation in the stimulation group proceeded more rapidly. For Tb.N, the non-stimulation group tended to show a higher value than that in the stimulation group. For Tb.Sp, the stimulation group showed a lower value than that in the non-stimulation group. These results show that increases in bone volume could occur in the future.

Secretion of a factor that contributes to bone metabolism from muscle could be induced owing to stimulation. Thus, we measured expression of BMP-2 and IL-1β. BMP-2 belongs to the transforming growth factor-β superfamily and induces osteoblast differentiation strongly.^11^ IL-1β is known to promote bone absorption strongly by stimulating osteoclasts.^12^ For expression of BMP-2 and IL-1β, the stimulation group showed significantly higher values than those of the non-stimulation group, suggesting that contraction of skeletal muscle was promoted and bone formation occurred more actively. This result is in accordance with that in a study by Schwarz and colleagues that suggested that osteoplasty must be strengthened by incorporating machine stimulation in BMP-2 treatment.^13^

We also measured expression of the cytokines IL-6 and IL-15, which are secreted along with activation of skeletal muscle.^14,15^ The stimulation group showed significantly higher expression of IL-6 and IL-15 than those in the non-stimulation group.

Fong and Tapscott suggested that MyoD can change most types of cells into muscle cells.^16^ In the present study, the stimulation group showed significantly higher MyoD expression than that in the non-stimulation group. This result suggests that our equipment could provide a sufficient stimulus to cause muscle contraction to induce muscle hypertrophy. IL-15 is a protein that can regulate muscle mass by inhibiting protein degradation and accelerating differentiation.^17,18^ Induction of IL-15 expression with our stimulation device affected muscular hypertrophy and, as a result, MyoD expression might have been confirmed. Our measurements of expression of these muscle biomarkers were consistent with that of a report^16–18^ on muscular hypertrophy. IL-15 has been reported to promote osteoclast formation by synergistic action with, for example, receptor activator of nuclear factor-kappa B ligand.^19^ In our study, the stimulation group showed significantly higher expression of IL-6 and IL-15 than those in the non-stimulation group. Thus, we can infer that bone absorption was promoted and bone metabolism was active. However, we did not find bone absorption to be promoted strongly, but instead, bone formation was promoted. Ishimi and co-workers stated that IL-6 is secreted from skeletal muscle, produced by osteoblasts, and induces bone absorption.^20^ Hence, we inferred that our stimulation equipment promoted contraction of skeletal muscle, that there was direct physical stimulation to bone *via* tendons, and that a substance was secreted from skeletal muscle that promoted bone formation instead of bone absorption. Osteomorphometry and measurement of protein expression in QFMs suggested that our stimulation equipment did not suppress bone absorption and promoted bone formation by a direct physical stimulus to the bone but, instead, promoted cytokine secretion by stimulating skeletal muscle and producing a humoral factor that activated bone metabolism. Such promotion of bone metabolism could be demonstrated, but a clear change in bone structure could not. In bone remodeling in humans, bone absorption occurs first, and then bone formation progresses. Bone formation occurs more gradually than bone absorption.^21^ Therefore, it is possible that the stimulation conditions over 10 weeks were too short to increase bone volume.

Our stimulation equipment was directed at lumbar vertebrae. In past many studies, the target has been the femur. However, upon treadmill exercise: (i) joint exercise is difficult for lumbar vertebrae compared with that in the thigh and lower leg; and (ii) direct stimulation through the muscles attached to lumbar vertebrae (iliopsoas muscles) or through muscle strengthening is challenging. Our newly developed equipment may be able to stimulate the entire body from a flat plate and, therefore, have a stimulatory effect on lumbar vertebrae.

When considering the development and clinical application of our stimulation equipment, the cardiovascular load may be lower compared than that on muscles because the type of stimulation is passive. Also, our stimulation equipment can be applied even if a person has a physical impediment by adjusting the stimulation strength. Nevertheless, the rotation speed, gyration radius, and size of our apparatus could hinder clinical application.

## Conclusions

With the novel stimulation equipment used in this research, we demonstrated cytokine secretion through stimulation of skeletal muscles. Our results suggest that cytokine secretion by QFMs carried a humoral factor throughout the body *via* the blood and blood vessels and acted on bone and various organs.
